# HuAbDiffusion: a discrete language diffusion model used for antibody humanization

**DOI:** 10.1093/bib/bbaf658

**Published:** 2025-12-10

**Authors:** Dongping Liu, Xiaohu Hao, Long Fan

**Affiliations:** Production and R&D Center I of LSS (Life Science Service), GenScript Biotech Corporation, No. 28, Yongxi Rd, Nanjing, Jiangsu 211100, China; Production and R&D Center I of LSS (Life Science Service), GenScript Biotech Corporation, No. 28, Yongxi Rd, Nanjing, Jiangsu 211100, China; Production and R&D Center I of LSS (Life Science Service), GenScript Biotech Corporation, No. 28, Yongxi Rd, Nanjing, Jiangsu 211100, China; Production and R&D Center I of LSS (Life Science Service), GenScript (Shanghai) Biotech Corporation, No. 186, Hedan Rd, Shanghai Municipality 200100, China

**Keywords:** antibody, humanization, diffusion model, generative model

## Abstract

The humanization of antibodies (Abs) remains one of the main pathways for therapeutic antibody development. With the advantages of diffusion models, here we present HuAbDiffusion, a discrete language **diffusion** model used for antibody humanization by generating humanized antibodies from scratch. HuAbDiffusion starts from three complementary determinant regions (CDRs) and finally generate whole V region sequences. The model was evaluated on 22 mAbs and compared with several existing methods, the test results show the effectiveness and better performance of the proposed model. Besides, the potential optimal humanized antibodies to be selected could be narrowed down to a reasonable level with the usage of pretrained language models. The most significant is that the binding affinity of the humanized antibody can be retained or even increased generated by HuAbDiffusion. The method can be reached out through our previous established YabXnization server at https://www.genscript.com/tools/yabxnization-service.

## Introduction

Since the first monoclonal antibody drug was approved by the FDA in 1986, the market size of therapeutic antibodies has shown the trend of exponential growth [1]. Although in vitro directed evolution technology has become mature, cost-effective, and could better control antigen presentation [2], more and more biotechnology companies still tend to obtain new antibodies by isolating antibodies from patients [3, 4] or by immunizing genetically modified animals [5, 6]. So far, most therapeutic antibodies were developed from animal immunity [7], as antibodies evolved by the immune system typically have higher potential for development compared to in vitro evolution [8, 9]. However, antibodies produced in animal models often cause human intolerance*—*i.e. the immunogenic responses. Immunogenic reactions may have negative impacts on drug safety or pharmacokinetic properties, and produce neutralizing antibodies, leading to the loss of efficacy [10]. In order to eliminate or reduce the immunogenic reactions of therapeutic antibodies, various techniques have been developed to optimize the animal-sourced antibodies, including chimeric antibodies [11] and antibody humanization [12]. The former refers to the binding of the variable domain (V region) of animal-sourced antibodies with the constant region (C region) of human antibodies, while the latter involves grafting the complementary determining region (CDR) of animal-sourced antibodies onto the framework region (FR) of human antibodies. This procedure greatly reduces the possibility of immunogenicity caused by antibodies from animals. Due to the lower immunogenicity exhibited by more human antibody amino acids [13], humanization of animal antibody sequences remains one of the main pathways for therapeutic antibody development [7], accounting for 50% of the total number of commercialized therapeutic antibodies [14]. In addition, the humanized antibodies are expected to maintain the binding affinity for the target antigen without a significant decrease compared to the original animal-sourced antibody.

Traditional humanization of antibodies mainly relies on expert knowledge and experience in antibody development, which needs repeated experiments and is time consuming with low success rate [15]. Although some computational progress has been proposed, these schemes still face some limitations, e.g. they need manual input [16] and construction of the three-dimensional structures of the corresponding antibodies [17]. Fortunately, with the efforts of artificial intelligence, it has become possible to apply machine learning and deep learning methods for antibody humanization. Hu-mAb [18] is a random forest based model proposed for discriminating human and no-human antibody variable domain sequences. It can be also used for suggesting mutations of the antibody sequences to reduce their immunogenicity, since its output scores exhibit a negative relationship with the experimental immunogenicity of existing antibody therapeutics. On a set of therapeutic antibodies with known precursor sequences, the mutations suggested by Hu-mAb showed significant overlap with those deduced experimentally. BioPhi [19] is an open-source platform presented by Merck in 2022, which provides novel method for humanization (Sapiens) and humanness evaluation (OASis). On an *in silico* humanization benchmark, Sapiens produced sequences at scale and achieved comparable results to that of human experts produced. Abnativ [20] is a deep learning based tool for assessing the antibody nativeness which is the likelihood of a given antibody belongs to the distribution of immune-system-derived human antibodies. It provides an interpretable score which reflects the immunogenicity, and a residue-level profile which can guide the antibody engineering to distinguish the suitable humanized antibodies from the immune-system-derived ones.

**Figure 1:**
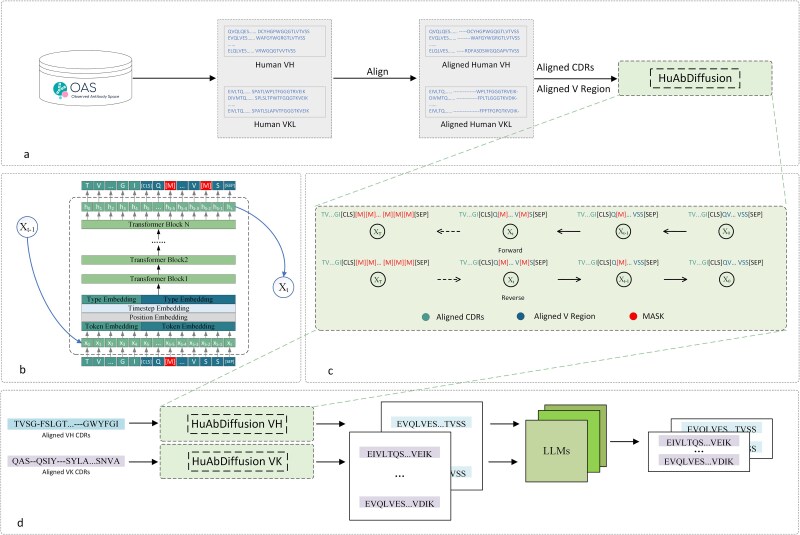
The overall process of HuAbDiffusion in conducting the humanization. (a) Collected data and preprocess of the data. (b) Backbone of HuAbDiffusion. (c) Forward and reverse process in HuAbDiffusion. (d) Filter the generated variant sequences through pretrained large language model.

In this study, we presented HuAbDiffusion, a generative diffusion model for humanization of heterologous antibodies at the sequence level. The model utilizes diffusion model framework to gradually mask the V region sequence under CDRs conditions, and learns the long sequence dependency relationship between CDRs and FRs in forward process, thus achieving antibody humanization in reverse process. Then several candidates would be generated in total. Further, the large number of the candidate humanized sequences can be filtered with the use of pretrained large language models. The model was evaluated on 22 mAbs and compared with several existing methods, the test results show the effectiveness and better performance of the proposed model. The overall process of HuAbDiffusion in conducting the humanization is depicted in Fig. 1.

The main contributions of this work can be summarized as follows:

- HuAbDiffusion is a generative model which combines the discrete diffusion model and Transformer, and applied to antibody humanization, which can generate whole humanized antibody V region sequences according to the given CDRs.
- HuAbDiffusion automatically selects mutation sites in the FR regions, which means the totally new sequence motif could be generated even they are not present in the realistic human antibodies, and the sequence diversity improved meanwhile.
- Selection problem of the candidate humanized antibodies was solved to a certain degree by pretrained large language model.

## Materials and Methods

### Training dataset

The training dataset was randomly extracted from the Observed Antibody Space (OAS) [21], which contains 2 million of unpaired human heavy chain sequences, 2 million of unpaired human kappa chain sequences and 2 million of human lambda chain sequences. All extracted antibody sequences were aligned under the AHo numbering scheme [22] and marked with North CDR definition [23] by ANARCI software [24], resulting in aligned V region sequences of length 149. The three CDRs in the aligned V regions are connected sequentially to form a CDRs region sequence with length 64 and 67 for heavy chain and light chain, respectively.

### Testing dataset

For clearly showing the performance of the constructed model, 22 therapeutic antibodies already used for clinical treatment in the SAbDab [14] database were collected as ground truth samples for model evaluation. The corresponding precursor sequences of the 22 antibodies were manually collected from patents and papers. The precursor and humanized sequences of the 22 selected antibodies are summarized in Table S8 in the **Supplementary Information**, **SI**.

### Diffusion model

Diffusion Model [25] has sparked a surge in protein engineering in recent years, including protein sequence generation [26–28], protein structure generation [29, 30] and collaborative generation of sequences along with structures [31]. Diffusion models are a class of latent variable models for generation, and they are consisting of a forward noising process and a reverse denoising process. Continuous domains and discrete domain correspond to different processes.

In continuous domains, given a sample ${x}_0\sim q\left({x}_0\right)$, a series of latent variable ${x}_1,{x}_2,\cdots{x}_t$ are generated by a Markov chain by adding a small Gaussian noise to the sample, described as:


$$ q\left({x}_t|{x}_{t-1}\right)=\mathcal{N}\left({x}_t;\sqrt{1-{\beta}_t}{x}_{t-1},{\beta}_tI\right), $$

where ${\left\{{\beta}_t\in \left(0,1\right)\right\}}_{t=1}^T$ is a noise schedule that controls how much noise is added at each step. Diffusion model learns the distribution of noise added to ${x}_0$ until ${x}_0$ become pure noise, which is called forward process, also known as noise adding process. Then, the diffusion model executes the distribution of noise learned in the forward process, restoring a given pure noise to an entity (such as a sequence, an image and so on) step by step according to following equation:


$$ {p}_{\theta}\left({x}_{t-1}|{x}_t\right)=\mathcal{N}\left({x}_{t-1};{\mu}_0\left({x}_t,t\right),{\sum}_{\theta}\left({x}_t,t\right)\right), $$

where $\theta$ is what we learn from the backbone model, such as U-Net [32] in DDPMs [25].

However, diffusion models with continuous variables are not suitable for nature language processing (NLP) tasks [33, 34], as unlike pixel values in images, sequences in NLP are typically represented as discrete variables. In discrete domain, each token is represented by a scalar discrete random variable with *K* categories. The forward process can be represented as:


$$ q\left({x}_t|{x}_{t-1}\right)= Cat\left({x}_t;p={x}_{t-1}{Q}_t\right), $$

where $Cat\left(x;p\right)$ is categorical distribution and ${Q}_t$ is transition probabilities metric. Accordingly, the reverse process can be described as following equations:


$$ q\left({x}_t|{x}_0\right)= Cat\left({x}_t:p={x}_0{\overline{Q}}_t\right), $$

$$ {\overline{Q}}_t={Q}_1{Q}_2\cdots{Q}_t, $$

\begin{align*} q\left({x}_{t-1}|{x}_t,{x}_0\right)&=\frac{q\left({x}_t|{x}_{t-1},{x}_0\right)q\left({x}_{t-1}|{x}_0\right)}{q\left({x}_t|{x}_0\right)}\nonumber\\&= Cat\left({x}_{t-1};p=\frac{x_t{Q}_t^T\odot{x}_0{\overline{Q}}_{t-1}}{x_0{\overline{Q}}_t{x}_t^T}\right). \end{align*}

### HuAbDiffusion architecture

Considering that only FR regions are modified in the general antibody humanization process, a CDRs-conditional generative diffusion model was proposed to keep CDRs always visible during the forward and reverse process without explicit CDR constraint. Specifically, the forward process of adding noise can be described as:


$$ q\left({x}_t|{x}_{t-1},c\right)= Cat\left({x}_t;p={x}_{t-1}{Q}_t\right). $$

The scheme of the diffusion part is on the basis of D3PM [33] and DiffusionBert [35], both generative masked language models based on discrete diffusion models, which combines the strengths of language models and diffusion models.

We can obtain the reverse process:


$$ q\left({x}_{t-1}|{x}_t,{x}_0,c\right)=\frac{q\left({x}_t|{x}_{t-1},{x}_0,c\right)q\left({x}_{t-1}|{x}_0,c\right)}{q\left({x}_t|{x}_0,c\right)}, $$

where ${\overline{Q}}_t={Q}_1{Q}_2\cdots{Q}_t$*.*where *c* is defined according to the CDRs in the current antibody sequence to be humanized.

The backbone model of HuAbDiffusion is consisted of 12 layers of Transformer blocks which contain 16 attention heads with hidden size of 1024. It is worth noting that in order to match the diffusion model, each Transformer block has been modified by embedding time into itself. Firstly, sample sequences are embedded by an embedding layer; Secondly, a position embedding layer is used to add positional information of tokens in the sequence, where CDRs and V region are considered as a whole sequence for adding positional information; Then, a type layer is added to distinguish the CDRs and V region in the whole sequence, where “0”s are added to the CDR positions and “1”s are added to the V region positions; Finally, the embedding can be input into 12 Transformer layers, and the logit output can be obtained. The architecture of HuAbDiffusion is depicted in Fig. 1b and c.

### Training process

The training process contains froward and reverse processes. The input is the combined sequence of the aligned CDRs as prefix and the entire aligned V region, which with a separator between them to distinguish their spatial positions. During the froward process, noise will be gradually added to the V region, and the corresponding positions will be replaced with the mask token which defined in vocabulary in discrete domain. When time step $t=T$, whole V region positions are masked, which represents pure noise sequence. What to be noted is that the CDRs region remains unchanged from $t=0\ to\ T$, which means only the V regions sequence undergoes a noising process, while the aligned CDRs sequence is only used for encoding the backbone and not involved in the diffusion process.

The model then embeds each input into a tensor with dimension of $149\times 26$, where 26 refers to the length of the vocabulary which contains 20 standard amino acids tokens and six other tokens such as gap symbols(−), start symbol ([CLS]), fill symbol ([PAD]), segmentation symbol ([SEP]), mask symbol ([MASK]) and unknown symbol ([UNK]) tokens. The CDR embedding feature ${E}_i^c\in{\mathbb{R}}^{1\times{L}_c\times d}$ for the *i*-th CDR (*c*) after embedding can be described as:


$$ {E}_i^c={s}_i^c\bullet{W}_E^c, $$

where ${s}_i^c\in{\mathbb{R}}^{1\times{L}_c}$ represents the encoded input sequence. For a batch input with batch size = *b*, ${s}^c\in{\mathbb{R}}^{b\times{L}_c}$. And V region features ${E}_i^v\in{\mathbb{R}}^{1\times{L}_v\times d}$ for the *i*-th V region along with [CLS] and [SEP] ([CLS] serves as the annotation at the input V region beginning and to distinguish between CDRs and V region of the input whole sequence. [SEP] is added at the end of the input to indicate the end.) can be described as:


$$ {E}_i^v= Cat\left(\left[ CLS\right],{s}_i^v,\left[ SEP\right]\right)\bullet{W}_E^v. $$

Then, position features (${P}_i^{cv}\in{\mathbb{R}}^{L_c+{L}_v}$) and segmentation features (${S}_i^{cv}\in{\mathbb{R}}^{L_c+{L}_v}$) are added to the entire input features:


$$ {E}_i^o= Cat\left({E}_i^c+{E}_i^v\right)+{P}_i^{cv}+{S}_i^{cv}. $$

The embedded tensor ${E}_i^o\in{\mathbb{R}}^{\left({L}_c+{L}_v\right)\times d}$ is then input to the Transformer blocks, and the output of the Transformer blocks can be described as:


$$ {z}_i^o= Dropout\left({Cat}_{k\in 1,2\dots H}\left( Softmax\left(\frac{q_i^{k,o}\bullet{\left({k}_i^{k,o}\right)}^T}{\sqrt{d_k}}\right)\bullet{v}_i^{k,o}\right)\right), $$

$$ {q}_i^{k,o}={W}_Q^{k,o} Norm\left({E}_i^o\right), $$

$$ {k}_i^{k,o}={W}_K^{k,o} Norm\left({E}_i^o\right), $$

$$ {v}_i^{k,o}={W}_V^{k,o} Norm\left({E}_i^o\right), $$

where ${q}_i^{k,o}$, ${k}_i^{k,o}$, and ${v}_i^{k,o}$ represents query, key and value in multihead attention, respectively. The ${z}_i^o$ is then returns to the starts of the diffusion process, recycling until the diffusion step (set to 512 in this study) goes over. After 512 steps, the entire V region sequence is completely masked.

The reverse process starts from the CDRs sequence which has been input to the forward process since the CDRs region is kept in the diffusion process. This corresponds to the general process of antibody humanization operation, which involves specifying CDRs and allowing the model to generate amino acids sequences in FR regions. When generating the sequence, the model requires the aligned CDRs with a masked sequence which length is 149. After 512 steps, the complete V region antibody sequence is generated. In addition, sampling temperature is introduced to control the quality of the sampled sequence. The sampling temperatures are set to 0.7 and 0.65 for heavy and light chains, respectively.

### Variants ranking

With the HuAbDiffusion model has been trained, a series of humanized sequences (variants) could be generated. How to get the variants with high binding affinity from them is also a critical problem. Previous works demonstrated that log-likelihood scores from state-of-the-art pretrained large language models for antibodies or proteins, e.g. ESM, correlate strongly with experimentally measured binding affinities [36, 37]. In such works, the authors used large language models or other generative models to evolve human antibodies, or suggesting mutations, according to the indication of log-likelihood score.

In this study, serval pretrained large language models including ESM-1b [38] and ESM-1v [39](ESM-1v1, ESM-1v2, ESM-1v3, ESM-1v4, and ESM-1v5) were untiled to calculate log-likelihood scores for ranking the variants. For each variant, the total log-likelihood score calculated at each position maps for ranking the variants. Here, we defined log-likelihood scores (*LL*) according to the following formula:


$$ LL={\sum}_{j=1}^m\mathit{\log}{P}_j\left({s}_j|M\right), $$

where *M* represents the large language models, *s* represents each token of the variant sequence. The variant with higher score ranks higher, and is more likely to be the candidate humanized sequence.

### Model implementation

HuAbDiffusion was implemented in Python 3.8 and run in an Intel(R) Xeon(R) Bronze 3206R machine with 256 GB RAM, 1.90 GHz CPU, Nvidia A100 GPU, and 64-bit Ubuntu Sever 20.04 operating system. The whole network was borrowed from the Python based PyTorch (version 2.1) package. The model was trained with batch size of 64 by AdamW optimizer with learning rate of $3E-5$.

### *In silico* model evaluation

To assess the quality of the humanized sequences generated by all comparison models, sequence-based and 3D structure-based metrics were employed:

1. The pLDDT was first proposed in AlphaFold2 [40] for self-assessment of prediction accuracy. By calculating the intrinsic distance of the predicted structure, the credibility of each residue in the predicted structure can be determined in the absence of natural structures. Considering computational efficiency, Chai [41] was used to compute the 3D structures of the ground truth samples and the humanized sequences generated by the models.

2. The scPerplexity [26, 27] was introduced to estimate the quality and reliability of generated sequence. For ground truth samples and generated sequences, ProteinMPNN [42] was used to reverse computed 3D structures into sequences and then calculate the scPerplexity.

3. The TMScore was first proposed by Zhang [43] to assess the quality of protein structure templates and predicted full-length models. The TMScores were calculated between the 3D structures of ground truth samples and the 3D structures of their corresponding humanized antibody sequences.

4. The RMSD (Root Mean Square Deviation) scores were also calculated between the 3D structures of ground truth samples and the 3D structures of their corresponding humanized antibody sequences on Vernier zone residues [44].

## Results

In this section, we first introduce the performance of HuAbDiffusion on the test dataset (22 samples) through *in silico* analysis, and then describe the validation on three humanized antibodies through wet laboratory experiments. Three state-of-the-art methods, Abnativ, BioPhi and Hu-mAb were used for comparison. Note that these three models only produce one sequence per chain type while HuAbDiffusion give a batch of humanized sequences. Therefore, the comparison was conducted on two aspects: the one sequence generated by the baseline method between (1) the overall performance of the batch of humanized antibodies generated by HuAbDiffusion, and (2) the best one in the batch.

### Variants ranking results

**Figure 2:**
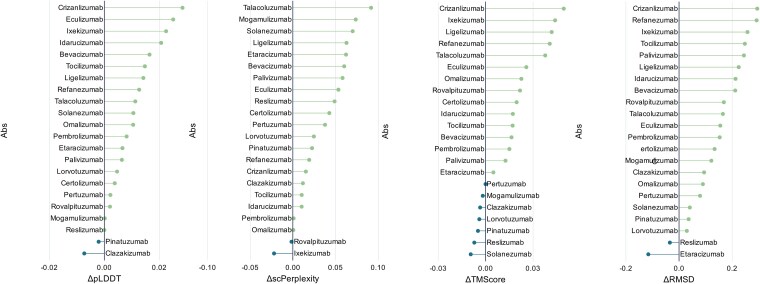
Ranking results on all 22 testing samples. Note that lower scPerplexity and RMSD are better, so scPerplexity and RMSD are calculated by subtracting the average before ranking from the average after ranking, while pLDDT and TMScore are calculated by subtracting the average before ranking from the average after ranking. The green dots indicate better results after ranking, while the blue dots indicate the opposite.

The top 10 variants in the generated batch sequences were selected by the log-likelihood scores for each testing sample, and their pLDDT, scPerplexity, TMScore and RMSD were calculated accordingly. Compared to the pLDDT, scPerplexity, TMScore and RMSD that calculated from the whole batch sequences, majority of the samples have an increase on these indicators. As shown in Fig. 2, the green and blue lines with an end dot represent increase and decrease on the performance indicators, respectively. On the three indicators of pLDDT, scPerplexity and RMSD, 20 out of 22 samples show an improvement. In terms of TMScore, 15 samples show an improvement, five samples show a decrease, and two samples show almost no change. We assume that the antibodies with better *in silico* indicators are more potential to be the candidate humanized variants.

**Figure 3:**
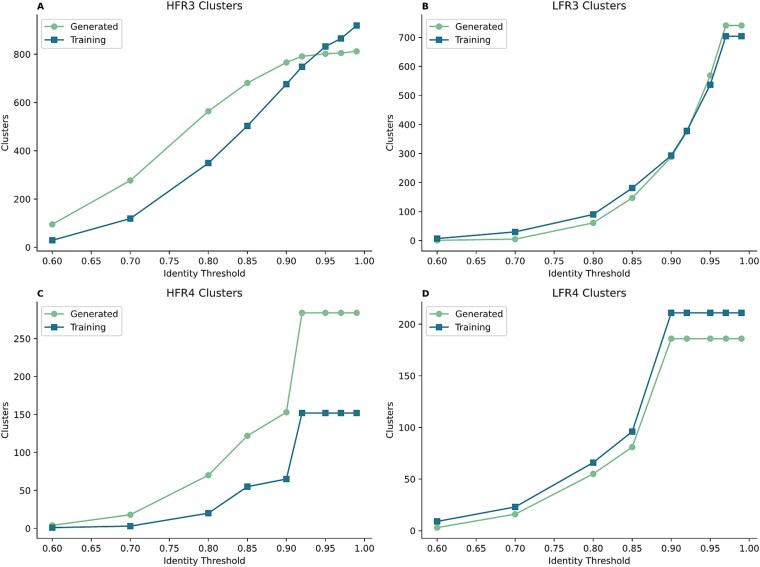
Clustering analysis of generated FR3 and FR4 sequences. The two graphs on the left side of the figure show the results of heavy chains, while the graphs on the right side show the results of light chains.

### Comparison on *in silico* model evaluation

As mentioned before, the comparison was conducted on two aspects: the one sequence generated by the baseline method between (1) the overall performance of the batch of humanized antibodies generated by HuAbDiffusion, and (2) the best one in the batch. Specifically, (1) calculates the average of all variants of these 22 antibodies (marked with “overall mean” in the Tables 1 and 2), (2) calculates the average of the best indicator value of the 22 antibodies (annotated “mean of the best” in Tables 1 and 2).

**Table 1:** Performance comparison on heavy chains.

**VH**	**pLDDT (↑)**	**scPerplexity (↓)**	**TMScore (↑)**	**RMSD (↓)**
HuAbDiffusion (overall mean)	**0.9422**	**1.2581**	0.9486	0.3664
HuAbDiffusion (mean of the best)	**0.9517**	**1.1768**	0.9626	**0.2613**
Abnativ	0.9328	1.3793	0.9737	0.3042
BioPhi	0.9366	1.3553	0.9753	0.5304
Hu-mAb	0.9333	1.4955	**0.9810**	0.6246

**Table 2:** Performance comparison on light chains.

**VKL**	**pLDDT (↑)**	**scPerplexity (↓)**	**TMScore (↑)**	**RMSD (↓)**
HuAbDiffusion (overall mean)	0.9570	1.2561	0.9847	0.2434
HuAbDiffusion (mean of best)	**0.9649**	**1.1775**	0.9917	**0.0865**
Abnativ	0.9590	1.2719	**0.9929**	0.1521
BioPhi	0.9613	1.2339	0.9904	0.1229
Hu-mAb	0.9572	1.3335	0.9922	0.1534

Under calculation method (2), for the heavy chain, HuAbDiffusion achieves 0.9517, 1.1768, and 0.2613 in terms of pLDDT, scPerplexity, and RMSD, respectively, all of which represent the best performance among the comparison methods. For the light chain, HuAbDiffusion attains 0.9649, 1.1775, and 0.0865 for the same metrics, again outperforming all other methods. These results demonstrate that HuAbDiffusion is capable of generating high-quality candidate humanized sequences. In addition, the RMSD results indicate that HuAbDiffusion effectively preserves the structural integrity of Vernier zone residues, which may help mitigate the affinity loss typically associated with the humanization process. The relatively unsatisfactory results for TMScore may be attributed to limitations in the predicted three-dimensional structures, since all variants are compared against the ground-truth structure we defined. Under calculation method (1), the performance of HuAbDiffusion is somewhat weaker yet still acceptable, which is mainly due to the small proportion of poorly performing individuals in the batch. Taken together, these findings indicate that selecting candidate humanized sequences based on ranking is a reasonable strategy.

### Diversity analysis of generated framework region sequences

To rigorously assess the diversity of model-generated FR sequences, we randomly sampled 1000 sequences from the training dataset and employed their CDRs to generate humanized antibodies. The resulting FRs were subjected to hierarchical clustering across a range of sequence identity thresholds (Fig. 3). For detailed examination, we focused on two representative FRs: FR3, generally the longest and least conserved framework segment, and FR4, which is shorter and typically more conserved. The clustering analysis revealed that the diversity of heavy chain FR sequences was modestly higher than that of the training set, whereas the diversity of light chain FR sequences remained largely comparable. Consistently across both chains, FR3 exhibited markedly greater diversity than FR4. Furthermore, the number of clusters increased monotonically with higher thresholds, closely mirroring the trend observed in the training data. Collectively, these findings demonstrate that the model not only faithfully captures the natural diversity distribution of antibody FRs but also introduces an additional degree of variability, particularly within the heavy chain, thereby balancing biological fidelity with generative innovation.

### Real-world applications

#### Test data

For further validating the effectiveness of the proposed HuAbDiffusion, three animal-sourced antibodies were selected to do the humanization, namely, clone 13, 16 and 38. Same preprocess with the training process was applied to the three antibodies and input to the trained HuAbDiffusion model for getting the top 1 candidate. The humanness analysis and verification of generation were conducted first, and the selected candidates were then expressed, and their binding affinity were tested through indirect enzyme-linked immunosorbent assay (ELISA).

#### Comparison on humanness evaluation

**Figure 4:**
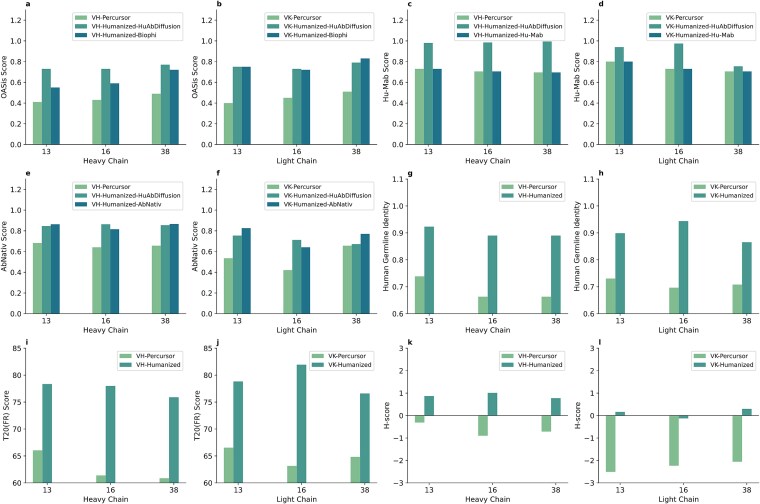
Humanness evaluation results. In each subgraph of (a–f), the scores are from the precursor, HuAbDiffusion, and the scoring model itself. In the subgraph of (g–l), scores are from the precursor and HuAbDiffusion.

Humanness is the degree of similarity between antibodies and those from humans. BioPhi, Hu-mAb and Abnativ were used for evaluating the humanness of the generated sequences and also used for comparison. Furthermore, Human Germline Identity, T20 Score [45] (only considering FR region) and H-Score [46] were also calculated, similar to HuDiff-Ab [47]. The humanness evaluation results are summarized in Fig. 4. In terms of OASis Identity score (Biophi), the humanness score of humanized variants versus that of its precursor of clone 13 (73% versus 41% on heavy chain, 75% versus 40% on light chain), clone 16 (73% versus 43% on heavy chain, 73% versus 45% on light chain), and clone 38(77% versus 49% on heavy chain, 79% versus 51% on light chain) show the increase on both light and heavy chains. The Hu-Mab score of heavy and light chains of clone 16 equal to 0.985 and 0.975, respectively. On the aspect of Abnativ score, the scores of humanized sequences of clone 13, 16 and 38 are 0.85, 0.86 and 0.85, respectively on heavy chains; the scores of humanized sequences of clone 13, 16 and 39 are 0.75, 0.71 and 0.67, respectively on light chains. In the other three indicators (Human Germline Identity, T20 Score and H-Scores), the scores of humanized antibodies are higher than those of precursors, both on the heavy and light chains.

The humanness evaluation results show the effectiveness of HuAbDiffusion in conducting the humanization operation, especially in eliminating the immunogenicity.

The further comparison of humanness on these three clones with BioPhi, Hu-mAb and Abnativ are also depicted in Fig. 4. In terms of Hu-Mab score, HuAbDiffusion achieves higher score than that of Hu-Mab on all three clones. In terms of the OASis Identity score, HuAbDiffusion achieves higher score than that of Biophi on clone 13 and 16, while slightly lower on light chain of clone 38, while the difference is not significant. In terms of AbNativ score, HuAbDiffusion achieves similar results than that of AbNativ. The results indicate that the FR regions generated by HuAbDiffusion for the three clones are close to those of human.

#### Immunogenicity evaluation

The ideal humanized therapeutic antibody should exhibit minimal immunogenicity. Thus, we examined the relationship between model-derived scores and clinically observed immunogenic responses, which was assessed based on the presence of antidrug antibody (ADA) responses. ADAs data for 217 therapeutic antibodies were collected from previously published studies [18, 48]. The model outputs were quantified by the Evidence Lower Bound (ELBO) score.

**Figure 5:**
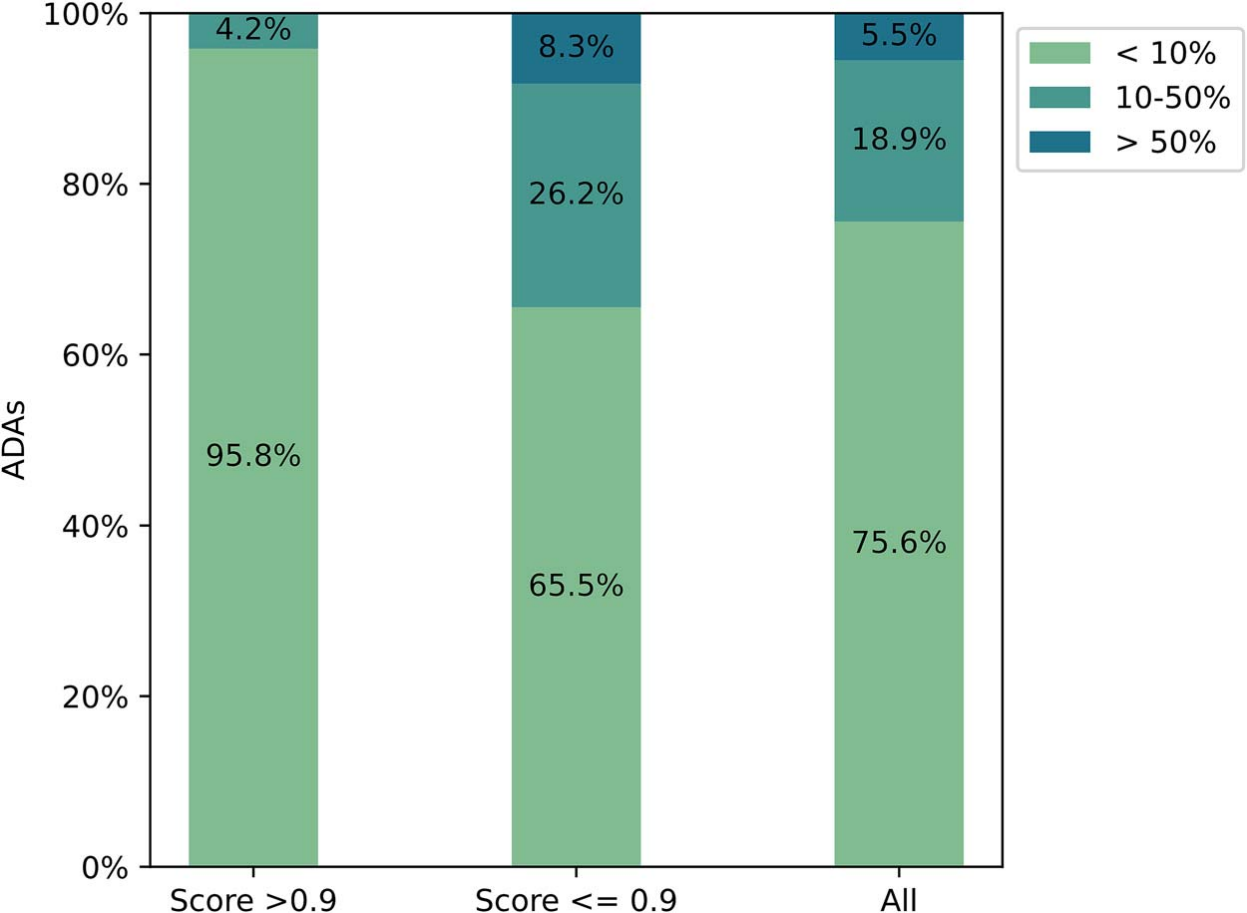
Association between normalized ELBO scores derived from the HuAbDiffusion model and clinically observed immunogenicity. Therapeutics were stratified into two groups based on the minimum normalized ELBO scores of the VH and VL/VK chains: scores above 0.9 (*n =* 72) and scores below 0.9 (*n =* 145). Immunogenicity was categorized into three levels according to ADA responses: high (>50%), moderate (10%–50%), and low (<10%).

To achieve a unified representation, the ELBO is normalized by min-max Normalization as the model’s score for input sequence. We evaluated the correlation between the percentage of ADA responses among 217 therapeutics and the minimum ELBO scores of the VH and VL/VK chains of the therapeutics as it is expected that the low score may determine the overall immunogenicity level. The ADAs dataset is divided into two groups based on a threshold of 0.9. As illustrated in Fig. 5, higher normalized ELBO scores were associated with reduced immunogenicity. In the group with scores above 0.9, 95.8% of sequences exhibited low ADAs incidence (<10%), and no sequences were associated with high ADAs levels (>50%). In contrast, sequences with scores below 0.9 tended to be more immunogenic. The proportion of sequences with low ADAs incidence decreased to 65.5%, those with moderate ADAs levels (10%–50%) increased to 26.2%, and sequences with high ADAs incidence (>50%) rose to 8.3%. Our analysis revealed that antibodies with higher normalized ELBO scores generally exhibited reduced immunogenicity, whereas those with lower scores were associated with increased ADA incidence. The trends observed in out model outputs mirror those reported in pervious investigations about ADA evaluation, including Hu-mAb [35] and Humatch [49].

#### Validation of generation process

Meaningful FR regions should be generated on the basis of human templates. However, if they are reproduced verbatim from the training data, they would likewise be meaningless, as this would simply reflect model overfitting. It means that the sequence similarity of generated FR regions should be neither 100%, nor a value that too low which indicates nonhomologous.

**Figure 6:**
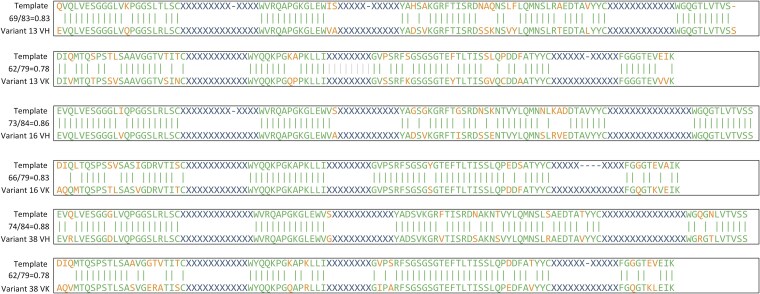
Validation results of generation process. The CDR regions are masked by “X”. The letters in green color refer to the identical amino acids between the generated FR regions and their corresponding template. The letters in orange color refer to the different ones.

Accordingly, the “Template Overlap Ratio” was defined for indicating the quality of FR regions generation. The “template” sequence is first to be found with the use of Blastp [50] program with the specified sequence (with whose CDR regions are masked by “X”) search against the training database. Then the number of identical amino acids between the specified sequence and the “template” sequence within the FR region was counted. Template Overlap Ratio of all three clones are shown in Table 3 and Fig. 6. Among all three clones, the highest and lowest scores are 0.88 (Variant 38 VH) and 0.78 (Variant 13 VK, Variant 38 VK), respectively. The maximum and minimum number of different positions compared to the corresponding “template” are 17 (Variant 13 VK, Variant 38 VK) and 10 (Variant 38 VH), respectively. Test results show that the FR regions generated by the HuAbDiffusion model are from scratch.

**Table 3:** Template overlap ratio of three clones.

	**13**		**16**		**38**	
	**VH**	**VK**	**VH**	**VK**	**VH**	**VK**
Template Overlap Ratio	0.83	0.78	0.86	0.83	0.88	0.78

### *In vitro* experiments

High humanness scores and good model evaluation metric indictors do not necessarily mean high affinity of the humanized antibodies. To further validate the effectiveness of humanized antibodies generated by HuAbDiffusion, *in vitro* binding affinity of clone 13, 16 and 38 and their corresponding chimeric antibodies were measurement by indirect Enzyme-Linked Immunosorbent Assay (ELISA).

To perform the experiment, coat a microtiter plate with a known concentration of antigen (1 μg/ml, 100 μL/well) using Phosphate Buffered Saline (PBS), pH 7.4. Prepare a series of antibody dilutions, starting from the original concentration and diluting 1:30 in each subsequent step, for a total of 12 steps. Incubate the antigen-coated wells with the different antibody dilutions. Following incubation, wash the wells with PBS containing 0.05% Tween-20 to remove any unbound antibodies. Subsequently, add a secondary antibody i.e. specific to the primary antibody and conjugated to an enzyme, such as Horseradish Peroxidase (HRP). Wash the wells again with PBS containing 0.05% Tween-20 to remove unbound secondary antibodies.

**Figure 7:**
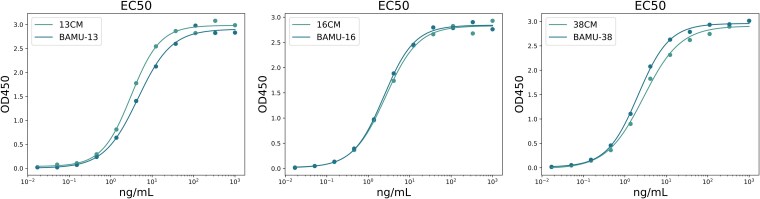
EC50 curve of three antibodies and their corresponding chimeric antibodies. From left to right are variant 13 (humanized antibody and chimeric antibody), variant 16(humanized antibody and chimeric antibody), and variant 38 (humanized antibody and chimeric antibody), respectively.

The ELISA test results are listed in Table 4 and shown in Fig. 7, which indicated that the binding affinities of the humanized antibodies for clone 16 and 38 are better than that of their corresponding chimeric antibodies (marked as 16CM, 38CM), for clone 13, the binding affinity of the humanized antibody is similar to its corresponding chimeric antibody (marked as 13CM). The Bio-Layer Interferometry (BLI) test has also conducted and summarized in Table S7 and Fig. S2, and similar conclusion can be obtained from the test results.

**Table 4:** EC50 values of three antibodies and their corresponding chimeric antibodies.

**mAbs**	**EC50(ng/ml)**	**If better than Chimeric Ab**
13CM	3.093	NO
BAMU-13	4.519	
16CM	2.610	YES
BAMU-16	2.374	
38CM	2.815	YES
BAMU-38	2.081	

**Figure 8:**
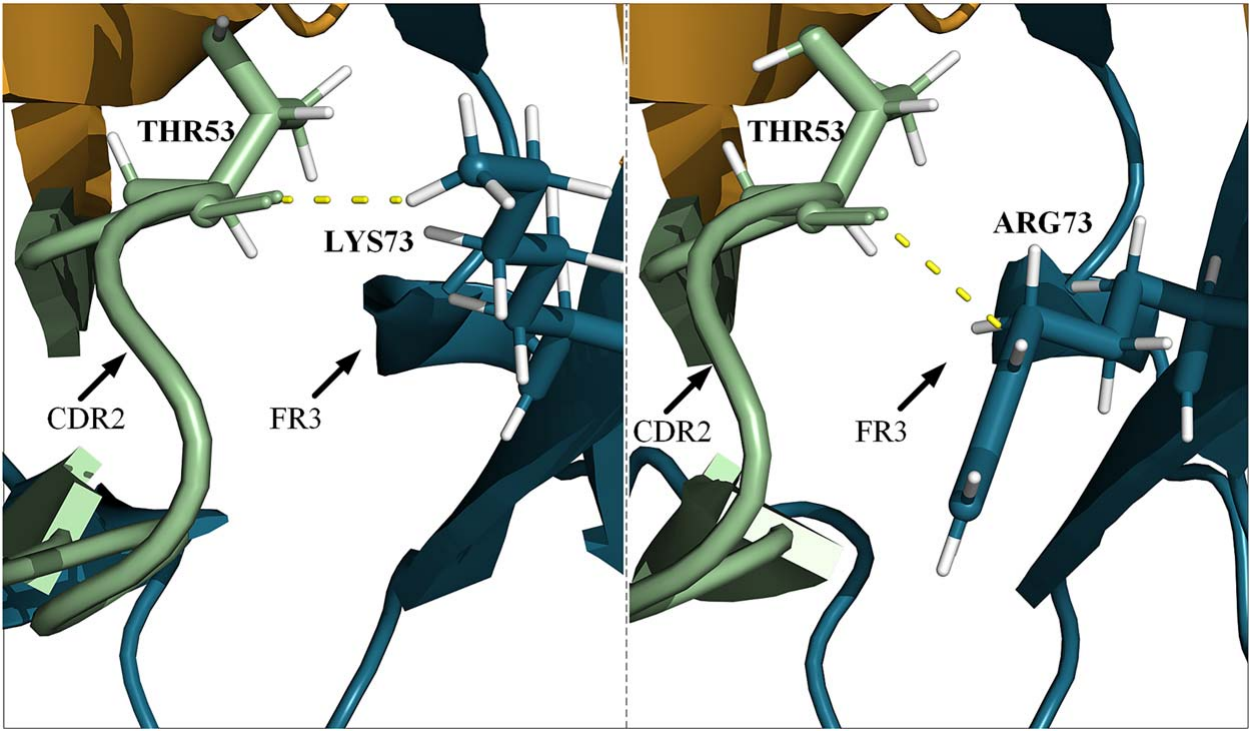
Crystal structures of 38CM and BAMU-38. Left: 38CM variant, THR53 in CDR2 and LYS73 in FR3 form a hydrogen bond. Right: BAMU-38 variant, LYS73 in FR3 is mutated to ARG73, which forms hydrogen bonds with THR53 in CDR2.

To further investigate the structural alterations affecting the binding affinity of variants, we used variant 38 as a case study to analyze the structural changes before (38CM) and after (BAMU-38) humanization. The crystal structures of 38CM (left panel, Fig. 8) and BAMU-38 (right panel, Fig. 8) were predicted by Chai. In the 38CM variant, hydrogen bonds were formed between THR53 in CDR2(green region) and LYS73 in FR3 (blue region), which contributed to the stability of the antibody structure. Upon humanization, the LYS73 residue in FR3 was replaced by ARG73, and the ARG73 formed hydrogen bonds with THR53 in CDR2. These hydrogen bonds between CDR2 and FR3 are known to be crucial in stabilizing the overall structure of antibodies [47, 51].

## Discussion

Certain bias between virtual evaluation indicators and wet-lab experimental results should be noticed in this work. In the real-world applications, test result showed that the performance of clone 38 was not satisfactory in terms of the in silico evaluation metrics. However, in vitro experiments showed that the binding affinity of the humanized antibody of clone 38 was even higher than that of its corresponding chimeric antibody. This discrepancy suggests that in silico evaluation metrics capture some complex relationships between antibody sequences and their attributions, while they don’t always align with the real binding affinity. Developing reliable methods for assessing biological responsible traits remains a challenge.

This template-free approach results in a diverse array of output variants. Although some sequences may not appear ideal, this does not prevent the discovery of exciting variants within the diverse results. Universal computational evaluation matrix could help to evaluate the results, but there might also be deviations to the experimental results. More broadly, establishing more accurate in silico evaluation metrics translating to higher developability are still challenges, and to some extent, even more critical than developing new antibody humanization models.

## Conclusion

In this work, HuAbDiffusion, a diffusion model used for antibody humanization with LLM models for ranking the humanized variants was presented. Test results showed that diffusion model combined with language model could generate high affinity humanized variants on the basis of given animal-sourced CDRs sequences. Compared to existing *in silico* solutions, only CDRs sequences of precursor antibodies is required, which eliminating the necessity of a human germline templates or three-dimensional structures. Besides, a test dataset including 22 therapeutic antibodies with their precursor sequences was constructed to evaluate the model performance, which can be used as a benchmark dataset.

**Key Points**

- Integrating diffusion model and language model to develop a novel computational approach named HuAbDiffusion for antibody humanization fully based sequences.
- Constructing an antibody drug dataset and comparing HuAbDiffusion with state-of-the-art antibody humanization methods on multiple indicators.
- Evaluating the performance of HuAbDiffusion by affinity assay.

## Supplementary Material

SupplementalMaterial_bbaf658

## Data Availability

The training data used in this work could be download from https://opig.stats.ox.ac.uk/webapps/oas/. And the web server for HuAbDiffusion is available at https://www.genscript.com/tools/yabxnization-service.
