# Supplementary material for: HuAbDiffusion: a discrete language diffusion model used for antibody humanization
Source: Brief Bioinform. 2025 Dec 10;26(6):bbaf658. doi: 10.1093/bib/bbaf658 (PMC12694429; doi:10.1093/bib/bbaf658)
Supplement: SupplementalMaterial_bbaf658 [file supplementalmaterial_bbaf658.docx]

# **Supplementary Information**

# of

# **HuAbDiffusion: A Discrete Language Diffusion Model Used for Antibody Humanization**

Dongping Liu^1^, Xiaohu Hao^1^, Long Fan^1,2^ *

1. Production and R&D Center I of LSS (Life Science Service), GenScript Biotech Corporation, No. 28, Yongxi Rd., Nanjing, 211100, Jiangsu, China

2. Production and R&D Center I of LSS (Life Science Service), GenScript (Shanghai) Biotech Corporation, No. 186, Hedan Rd., Shanghai, 200100, China

**Table S1.** OASis scores of three antibodies and their corresponding precursors. The results in parentheses are from Biophi.

| Name | Score | Metrics | Chain Type | Precursor or Humanized |
| --- | --- | --- | --- | --- |
| 13 | 0.41 | OASis | VH | Precursor |
| 13 | 0.73（0.55） | OASis | VH | Humanized |
| 13 | 0.40 | OASis | VK | Precursor |
| 13 | 0.75（0.75） | OASis | VK | Humanized |
| 16 | 0.43 | OASis | VH | Precursor |
| 16 | 0.73（0.59） | OASis | VH | Humanized |
| 16 | 0.45 | OASis | VK | Precursor |
| 16 | 0.73（0.72） | OASis | VK | Humanized |
| 38 | 0.49 | OASis | VH | Precursor |
| 38 | 0.77（0.72） | OASis | VH | Humanized |
| 38 | 0.51 | OASis | VK | Precursor |
| 38 | 0.79（0.83） | OASis | VK | Humanized |

**Table S2.** Hu-Mab scores of three antibodies and their corresponding precursors. The results in parentheses are from Hu-Mab.

| Name | Score | Metrics | Chain Type | Precursor or Humanized |
| --- | --- | --- | --- | --- |
| 13 | 0.730 | Hu-Mab | VH | Precursor |
| 13 | 0.980 (0.730) | Hu-Mab | VH | Humanized |
| 13 | 0.800 | Hu-Mab | VK | Precursor |
| 13 | 0.940 (0.800) | Hu-Mab | VK | Humanized |
| 16 | 0.705 | Hu-Mab | VH | Precursor |
| 16 | 0.985 (0.705) | Hu-Mab | VH | Humanized |
| 16 | 0.730 | Hu-Mab | VK | Precursor |
| 16 | 0.975 (0.730) | Hu-Mab | VK | Humanized |
| 38 | 0.695 | Hu-Mab | VH | Precursor |
| 38 | 0.995 (0.695) | Hu-Mab | VH | Humanized |
| 38 | 0.705 | Hu-Mab | VK | Precursor |
| 38 | 0.755 (0.705) | Hu-Mab | VK | Humanized |

**Table S3.** Hu-Mab scores of three antibodies and their corresponding precursors. The results in parentheses are from AbNativ.

| Name | Score | Metrics | Chain Type | Precursor or Humanized |
| --- | --- | --- | --- | --- |
| 13 | 0.6820 | AbNativ | VH | Precursor |
| 13 | 0.8455 (0.8632) | AbNativ | VH | Humanized |
| 13 | 0.5354 | AbNativ | VK | Precursor |
| 13 | 0.7532 (0.8254) | AbNativ | VK | Humanized |
| 16 | 0.6411 | AbNativ | VH | Precursor |
| 16 | 0.8637 (0.8155) | AbNativ | VH | Humanized |
| 16 | 0.4212 | AbNativ | VK | Precursor |
| 16 | 0.7122 (0.6411) | AbNativ | VK | Humanized |
| 38 | 0.6567 | AbNativ | VH | Precursor |
| 38 | 0.8540 (0.8661) | AbNativ | VH | Humanized |
| 38 | 0.4753 | AbNativ | VK | Precursor |
| 38 | 0.6715 (0.7699) | AbNativ | VK | Humanized |

**Table S4.** Human Germline Identity of three antibodies and their corresponding precursors.

| Name | Score | Metrics | Chain Type | Precursor or Humanized |
| --- | --- | --- | --- | --- |
| 13 | 0.7386 | Human Germline Identity | VH | Precursor |
| 13 | 0.9230 | Human Germline Identity | VH | Humanized |
| 13 | 0.7303 | Human Germline Identity | VK | Precursor |
| 13 | 0.8988 | Human Germline Identity | VK | Humanized |
| 16 | 0.6629 | Human Germline Identity | VH | Precursor |
| 16 | 0.8901 | Human Germline Identity | VH | Humanized |
| 16 | 0.6966 | Human Germline Identity | VK | Precursor |
| 16 | 0.9438 | Human Germline Identity | VK | Humanized |
| 38 | 0.6629 | Human Germline Identity | VH | Precursor |
| 38 | 0.8901 | Human Germline Identity | VH | Humanized |
| 38 | 0.7078 | Human Germline Identity | VK | Precursor |
| 38 | 0.8651 | Human Germline Identity | VK | Humanized |

**Table S5.** T20 (FR) Score of three antibodies and their corresponding precursors.

| Name | Score | Metrics | Chain Type | Precursor or Humanized |
| --- | --- | --- | --- | --- |
| 13 | 66.04 | T20 (FR) Score | VH | Precursor |
| 13 | 78.34 | T20 (FR) Score | VH | Humanized |
| 13 | 66.54 | T20 (FR) Score | VK | Precursor |
| 13 | 78.81 | T20 (FR) Score | VK | Humanized |
| 16 | 61.38 | T20 (FR) Score | VH | Precursor |
| 16 | 77.98 | T20 (FR) Score | VH | Humanized |
| 16 | 63.15 | T20 (FR) Score | VK | Precursor |
| 16 | 81.93 | T20 (FR) Score | VK | Humanized |
| 38 | 60.86 | T20 (FR) Score | VH | Precursor |
| 38 | 75.89 | T20 (FR) Score | VH | Humanized |
| 38 | 64.81 | T20 (FR) Score | VK | Precursor |
| 38 | 76.59 | T20 (FR) Score | VK | Humanized |

**Table S6.** H-Score of three antibodies and their corresponding precursors.

| Name | Score | Metrics | Chain Type | Precursor or Humanized |
| --- | --- | --- | --- | --- |
| 13 | -0.3100 | H-Score | VH | Precursor |
| 13 | 0.8670 | H-Score | VH | Humanized |
| 13 | -2.5090 | H-Score | VK | Precursor |
| 13 | 0.1610 | H-Score | VK | Humanized |
| 16 | -0.8970 | H-Score | VH | Precursor |
| 16 | 1.0100 | H-Score | VH | Humanized |
| 16 | -2.2350 | H-Score | VK | Precursor |
| 16 | -0.1260 | H-Score | VK | Humanized |
| 38 | -0.7120 | H-Score | VH | Precursor |
| 38 | 0.7760 | H-Score | VH | Humanized |
| 38 | -2.0580 | H-Score | VK | Precursor |
| 38 | 0.2950 | H-Score | VK | Humanized |

**Table S7.** Variant 13, 16 and 38 BLI results. The affinity of humanized antibodies BAMU-16 and BAMU-13 is higher than their corresponding antibodies (13CM and 16CM).

| Name | Rmax | KD (M) | Full R2 |
| --- | --- | --- | --- |
| 38CM | 0.6651 | <1.0E-11 | 0.9994 |
| BAMU-38 | 0.4692 | <1.0E-11 | 0.9988 |
| BAMU-16 | 0.3748 | 1.747E-10 | 0.9978 |
| BAMU-13 | 0.2291 | 3.600E-10 | 0.9971 |
| 16CM | 0.3740 | 6.329E-10 | 0.9984 |
| 13CM | 0.1114 | 6.716E-09 | 0.9667 |

**Table S8.** Sequence information of 22 antibodies, including precursor and humanized sequences

| **Name** | **sequence** |
| --- | --- |
| **Bevacizumab_VH_Precursor** | **EIQLVQSGPELKQPGETVRISCKASGYTFTNYGMNWVKQAPGKGLKWMGWINTYTGEPTYAADFKRRFTFSLETSASTAYLQISNLKNDDTATYFCAKYPHYYGSSHWYFDVWGAGTTVTVSS** |
| **Bevacizumab_VH_Humanized** | **EVQLVESGGGLVQPGGSLRLSCAASGYTFTNYGMNWVRQAPGKGLEWVGWINTYTGEPTYAADFKRRFTFSLDTSKSTAYLQMNSLRAEDTAVYYCAKYPHYYGSSHWYFDVWGQGTLVTVSS** |
| **Bevacizumab_VK_Precursor** | **DIQMTQTTSSLSASLGDRVIISCSASQDISNYLNWYQQKPDGTVKVLIYFTSSLHSGVPSRFSGSGSGTDYSLTISNLEPEDIATYYCQQYSTVPWTFGGGTKLEIK** |
| **Bevacizumab_VK_Humanized** | **DIQMTQSPSSLSASVGDRVTITCSASQDISNYLNWYQQKPGKAPKVLIYFTSSLHSGVPSRFSGSGSGTDFTLTISSLQPEDFATYYCQQYSTVPWTFGQGTKVEIK** |
| **Omalizumab_VH_Precursor** | **DVQLQESGPGLVKPSQSLSLACSVTGYSITSGYSWNWIRQFPGNKLEWMGSITYDGSSNYNPSLKNRISVTRDTSQNQFFLKLNSATAEDTATYYCARGSHYFGHWHFAVWGAGTTVTVSS** |
| **Omalizumab_VH_Humanized** | **EVQLVESGGGLVQPGGSLRLSCAVSGYSITSGYSWNWIRQAPGKGLEWVASITYDGSTNYADSVKGRFTISRDDSKNTFYLQMNSLRAEDTAVYYCARGSHYFGHWHFAVWGQGTLVTVSS** |
| **Omalizumab_VK_Precursor** | **DIQLTQSPASLAVSLGQRATISCKASQSVDYDGDSYMNWYQQKPGQPPILLIYAASYLGSEIPARFSGSGSGTDFTLNIHPVEEEDAATFYCQQSHEDPYTFGAGTKLEIK** |
| **Omalizumab_VK_Humanized** | **DIQLTQSPSSLSASVGDRVTITCRASQSVDYDGDSYMNWYQQKPGKAPKLLIYAASYLESGVPSRFSGSGSGTDFTLTISSLQPEDFATYYCQQSHEDPYTFGQGTKVEIK** |
| **Eculizumab_VH_Precursor** | **QVQLQQSGAELMKPGASVKMSCKATGYIFSNYWIQWIKQRPGHGLEWIGEILPGSGSTEYTENFKDKAAFTADTSSNTAYMQLSSLTSEDSAVYYCARYFFGSSPNWYFDVWGAGTTVTVSS** |
| **Eculizumab_VH_Humanized** | **QVQLVQSGAEVKKPGASVKVSCKASGYIFSNYWIQWVRQAPGQGLEWMGEILPGSGSTEYTENFKDRVTMTRDTSTSTVYMELSSLRSEDTAVYYCARYFFGSSPNWYFDVWGQGTLVTVSS** |
| **Eculizumab_VK_Precursor** | **DIQMTQSPASLSASVGETVTITCGASENIYGALNWYQRKQGKSPQLLIYGATNLADGMSSRFSGSGSGRQYYLKISSLHPDDVATYYCQNVLNTPLTFGAGTKLELK** |
| **Eculizumab_VK_Humanized** | **DIQMTQSPSSLSASVGDRVTITCGASENIYGALNWYQQKPGKAPKLLIYGATNLADGVPSRFSGSGSGTDFTLTISSLQPEDFATYYCQNVLNTPLTFGQGTKVEIK** |
| **Tocilizumab_VH_Precursor** | **DVQLQESGPVLVKPSQSLSLTCTVTGYSITSDHAWSWIRQFPGNKLEWMGYISYSGITTYNPSLKSRISITRDTSKNQFFLQLNSVTTGDTSTYYCARSLARTTAMDYWGQGTSVTVSS** |
| **Tocilizumab_VH_Humanized** | **QVQLQESGPGLVRPSQTLSLTCTVSGYSITSDHAWSWVRQPPGRGLEWIGYISYSGITTYNPSLKSRVTMLRDTSKNQFSLRLSSVTAADTAVYYCARSLARTTAMDYWGQGSLVTVSS** |
| **Tocilizumab_VK_Precursor** | **DIQMTQTTSSLSASLGDRVTISCRASQDISSYLNWYQQKPDGTIKLLIYYTSRLHSGVPSRFSGSGSGTDYSLTINNLEQEDIATYFCQQGNTLPYTFGGGTKLEIN** |
| **Tocilizumab_VK_Humanized** | **DIQMTQSPSSLSASVGDRVTITCRASQDISSYLNWYQQKPGKAPKLLIYYTSRLHSGVPSRFSGSGSGTDFTFTISSLQPEDIATYYCQQGNTLPYTFGQGTKVEIK** |
| **Pembrolizumab_VH_Precursor** | **QVQLQQPGAELVKPGTSVKLSCKASGYTFTNYYMYWVKQRPGQGLEWIGGINPSNGGTNFNEKFKNKATLTVDSSSSTTYMQLSSLTSEDSAVYYCTRRDYRFDMGFDYWGQGTTLTVSS** |
| **Pembrolizumab_VH_Humanized** | **QVQLVQSGVEVKKPGASVKVSCKASGYTFTNYYMYWVRQAPGQGLEWMGGINPSNGGTNFNEKFKNRVTLTTDSSTTTAYMELKSLQFDDTAVYYCARRDYRFDMGFDYWGQGTTVTVSS** |
| **Pembrolizumab_VK_Precursor** | **DIVLTQSPASLAVSLGQRAAISCRASKGVSTSGYSYLHWYQQKPGQSPKLLIYLASYLESGVPARFSGSGSGTDFTLNIHPVEEEDAATYYCQHSRDLPLTFGTGTKLELK** |
| **Pembrolizumab_VK_Humanized** | **EIVLTQSPATLSLSPGERATLSCRASKGVSTSGYSYLHWYQQKPGQAPRLLIYLASYLESGVPARFSGSGSGTDFTLTISSLEPEDFAVYYCQHSRDLPLTFGGGTKVEIK** |
| **Pertuzumab_VH_Precursor** | **EVQLQQSGPELVKPGTSVKISCKASGFTFTDYTMDWVKQSHGKSLEWIGDVNPNSGGSIYNQRFKGKASLTVDRSSRIVYMELRSLTFEDTAVYYCARNLGPSFYFDYWGQGTTLTVSS** |
| **Pertuzumab_VH_Humanized** | **EVQLVESGGGLVQPGGSLRLSCAASGFTFTDYTMDWVRQAPGKGLEWVADVNPNSGGSIYNQRFKGRFTLSVDRSKNTLYLQMNSLRAEDTAVYYCARNLGPSFYFDYWGQGTLVTVSS** |
| **Pertuzumab_VK_Precursor** | **DTVMTQSHKIMSTSVGDRVSITCKASQDVSIGVAWYQQRPGQSPKLLIYSASYRYTGVPDRFTGSGSGTDFTFTISSVQAEDLAVYYCQQYYIYPYTFGGGTKLEIK** |
| **Pertuzumab_VK_Humanized** | **DIQMTQSPSSLSASVGDRVTITCKASQDVSIGVAWYQQKPGKAPKLLIYSASYRYTGVPSRFSGSGSGTDFTLTISSLQPEDFATYYCQQYYIYPYTFGQGTKVEIK** |
| **Ixekizumab_VH_Precursor** | **QVQLQQSRPELVKPGASVKISCKASGYSFTDYNMNWVKQSNGKSLEWIGVINPNYGTTDYNQRFKGKATLTVDQSSRTAYMQLNSLTSEDSAVYYCVIYDYATGTGGYWGQGSPLTVSS** |
| **Ixekizumab_VH_Humanized** | **QVQLVQSGAEVKKPGSSVKVSCKASGYSFTDYHIHWVRQAPGQGLEWMGVINPMYGTTDYNQRFKGRVTITADESTSTAYMELSSLRSEDTAVYYCARYDYFTGTGVYWGQGTLVTVSS** |
| **Ixekizumab_VK_Precursor** | **DVVLTQTPLSLPVSLGDQASISCRSSQSLVHSNGNTYLHWYLQKPGQSPKLLIYKVSNRFSGVPDRFSGSGSGTDFTLKISRVEAEDLGVYFCSQSTHVPFTFGSGTKLEIK** |
| **Ixekizumab_VK_Humanized** | **DIVMTQTPLSLSVTPGQPASISCRSSRSLVHSRGNTYLHWYLQKPGQSPQLLIYKVSNRFIGVPDRFSGSGSGTDFTLKISRVEAEDVGVYYCSQSTHLPFTFGQGTKLEIK** |
| **Palivizumab_VH_Precursor** | **QVELQESGPGILQPSQTLSLTCSFSGFSLSTSGMSVGWIRQPSGEGLEWLADIWWDDKKDYNPSLKSRLTISKDTSSNQVFLKITGVDTADTATYYCARSMITNWYFDVWGAGTTVTVSS** |
| **Palivizumab_VH_Humanized** | **QVTLRESGPALVKPTQTLTLTCTFSGFSLSTSGMSVGWIRQPPGKALEWLADIWWDDKKDYNPSLKSRLTISKDTSKNQVVLKVTNMDPADTATYYCARSMITNWYFDVWGAGTTVTVSS** |
| **Palivizumab_VK_Precursor** | **DIQLTQSPAIMSASPGEKVTMTCSASSSVGYMHWYQQKSSTSPKLWIYDTSKLASGVPGRFSGSGSGNSYSLTISSIQAEDVATYYCFQGSGYPFTFGQGTKLEIK** |
| **Palivizumab_VK_Humanized** | **DIQMTQSPSTLSASVGDRVTITCKCQLSVGYMHWYQQKPGKAPKLLIYDTSKLASGVPSRFSGSGSGTEFTLTISSLQPDDFATYYCFQGSGYPFTFGGGTKLEIK** |
| **Certolizumab_VH_Precursor** | **QIQLVQSGPELKKPGETVKISCKASGYVFTDYGMNWVKQAPGKAFKWMGWINTYIGEPIYVDDFKGRFAFSLETSASTAFLQINNLKNEDTATYFCARGYRSYAMDYWGQGTSVTVSS** |
| **Certolizumab_VH_Humanized** | **EVQLVESGGGLVQPGGSLRLSCAASGYVFTDYGMNWVRQAPGKGLEWMGWINTYIGEPIYADSVKGRFTFSLDTSKSTAYLQMNSLRAEDTAVYYCARGYRSYAMDYWGQGTLVTVSS** |
| **Certolizumab_VK_Precursor** | **DIVMTQSQKFMSTSVGDRVSVTCKASQNVGTNVAWYQQKPGQSPKALIYSASFLYSGVPYRFTGSGSGTDFTLTISTVQSEDLAEYFCQQYNIYPLTFGAGTKLELK** |
| **Certolizumab_VK_Humanized** | **DIQMTQSPSSLSASVGDRVTITCKASQNVGTNVAWYQQKPGKAPKALIYSASFLYSGVPYRFSGSGSGTDFTLTISSLQPEDFATYYCQQYNIYPLTFGQGTKVEIK** |
| **Idarucizumab_VH_Precursor** | **QVQLEQSGPGLVAPSQRLSITCTVSGFSLTSYIVDWVRQSPGKGLEWLGVIWAGGSTGYNSALRSRLSITKSNSKSQVFLQMNSLQTDDTAIYYCASAAYYSYYNYDGFAYWGQGTLVTVSA** |
| **Idarucizumab_VH_Humanized** | **QVQLQESGPGLVKPSETLSLTCTVSGFSLTSYIVDWIRQPPGKGLEWIGVIWAGGSTGYNSALRSRVSITKDTSKNQFSLKLSSVTAADTAVYYCASAAYYSYYNYDGFAYWGQGTLVTVSS** |
| **Idarucizumab_VK_Precursor** | **DVVMTQTPLTLSVTIGQPASISCKSSQSLLYTNGKTYLYWLLQRPGQSPKRLIYLVSKLDSGVPDRFSGSGSGTDFTLKISRVEAEDVGIYYCLQSTHFPHTFGGGTKLEIK** |
| **Idarucizumab_VK_Humanized** | **DVVMTQSPLSLPVTLGQPASISCKSSQSLLYTDGKTYLYWFLQRPGQSPRRLIYLVSKLDSGVPDRFSGSGSGTDFTLKISRVEAEDVGVYYCLQSTHFPHTFGGGTKVEIK** |
| **Reslizumab_VH_Precursor** | **EVKLLESGGGLVQPSQTLSLTCTVSGLSLTSNSVNWIRQPPGKGLEWMGLIWSNGDTDYNSAIKSRLSISRDTSKSQVFLKMNSLQSEDTAMYFCAREYYGYFDYWGQGVMVTVSS** |
| **Reslizumab_VH_Humanized** | **EVQLVESGGGLVQPGGSLRLSCAVSGLSLTSNSVNWIRQAPGKGLEWVGLIWSNGDTDYNSAIKSRFTISRDTSKSTVYLQMNSLRAEDTAVYYCAREYYGYFDYWGQGTLVTVSS** |
| **Reslizumab_VK_Precursor** | **DIQMTQSPASLSASLGETISIECLASEGISSYLAWYQQKPGKSPQLLIYGANSLQTGVPSRFSGSGSATQYSLKISSMQPEDEGDYFCQQSYKFPNTFGAGTKLELK** |
| **Reslizumab_VK_Humanized** | **DIQMTQSPSSLSASVGDRVTITCLASEGISSYLAWYQQKPGKAPKLLIYGANSLQTGVPSRFSGSGSATDYTLTISSLQPEDFATYYCQQSYKFPNTFGQGTKVEVK** |
| **Solanezumab_VH_Precursor** | **EVKLVESGGGLVQPGGSLKLSCAVSGFTFSRYSMSWVRQTPEKRLELVAQINSVGNSTYYPDTVKGRFTISRDNAEYTLSLQMSGLRSDDTATYYCASGDYWGQGTTLTVSS** |
| **Solanezumab_VH_Humanized** | **EVQLVESGGGLVQPGGSLRLSCAASGFTFSRYSMSWVRQAPGKGLELVAQINSVGNSTYYPDTVKGRFTISRDNAKNTLYLQMNSLRAEDTAVYYCASGDYWGQGTLVTVSS** |
| **Solanezumab_VK_Precursor** | **DVVMTQTPLSLPVSLGDQASISCRSSQSLIYSDGNAYLHWFLQKPGQSPKLLIYKVSNRFSGVPDRFSGSGSGTDFTLKISRVETEDLGVYFCSQSTHVPWTFGGGTKLEIK** |
| **Solanezumab_VK_Humanized** | **DVVMTQSPLSLPVTLGQPASISCRSSQSLIYSDGNAYLHWFLQKPGQSPRLLIYKVSNRFSGVPDRFSGSGSGTDFTLKISRVEAEDVGVYYCSQSTHVPWTFGQGTKVEIK** |
| **Lorvotuzumab_VH_Precursor** | **DVQLVESGGGLVQPGGSRKLSCAASGFTFSSFGMHWVRQAPEKGLEWVAYISSGSFTIYHADTVKGRFTISRDNPKNTLFLQMTSLRAEDTAHYYCARMRKGYAMDYWGQGTTVTVSS** |
| **Lorvotuzumab_VH_Humanized** | **QVQLVESGGGVVQPGRSLRLSCAASGFTFSSFGMHWVRQAPGKGLEWVAYISSGSFTIYYADSVKGRFTISRDNSKNTLYLQMNSLRAEDTAVYYCARMRKGYAMDYWGQGTLVTVSS** |
| **Lorvotuzumab_VK_Precursor** | **DVLMTQTPLSLPVSLGDQASISCRSSQIIIHSDGNTYLEWFLQKPGQSPKLLIYKVSNRFSGVPDRFSGSGSGTDFTLMISRVEAEDLGVYYCFQGSHVPHTFGGGTKLEIK** |
| **Lorvotuzumab_VK_Humanized** | **DVVMTQSPLSLPVTLGQPASISCRSSQIIIHSDGNTYLEWFQQRPGQSPRRLIYKVSNRFSGVPDRFSGSGSGTDFTLKISRVEAEDVGVYYCFQGSHVPHTFGQGTKVEIK** |
| **Pinatuzumab_VH_Precursor** | **QVQLQQSGPELVKPGASVKISCKASGYEFSRSWMNWVKQRPGQGREWIGRIYPGDGDTNYSGKFKGKATLTADKSSSTAYMQLSSLTSVDSAVYFCARDGSSWDWYFDVWGAGTTVTVSS** |
| **Pinatuzumab_VH_Humanized** | **EVQLVESGGGLVQPGGSLRLSCAASGYEFSRSWMNWVRQAPGKGLEWVGRIYPGDGDTNYSGKFKGRFTISADTSKNTAYLQMNSLRAEDTAVYYCARDGSSWDWYFDVWGQGTLVTVSS** |
| **Pinatuzumab_VK_Precursor** | **DILMTQTPLSLPVSLGDQASISCRSSQSIVHSNGNTFLEWYLQKPGQSPKLLIYKVSNRFSGVPDRFSGSGSGTDFTLKISRVEAEDLGVYYCFQGSQFPYTFGGGTKVEIK** |
| **Pinatuzumab_VK_Humanized** | **DIQMTQSPSSLSASVGDRVTITCRSSQSIVHSVGNTFLEWYQQKPGKAPKLLIYKVSNRFSGVPSRFSGSGSGTDFTLTISSLQPEDFATYYCFQGSQFPYTFGQGTKVEIK** |
| **Etaracizumab_VH_Precursor** | **EVQLEESGGGLVKPGGSLKLSCAASGFAFSSYDMSWVRQIPEKRLEWVAKVSSGGGSTYYLDTVQGRFTISRDNAKNTLYLQMSSLNSEDTAMYYCARHNYGSFAYWGQGTLVTVSA** |
| **Etaracizumab_VH_Humanized** | **QVQLVESGGGVVQPGRSLRLSCAASGFTFSSYDMSWVRQAPGKGLEWVAKVSSGGGSTYYLDTVQGRFTISRDNSKNTLYLQMNSLRAEDTAVYYCARHLHGSFASWGQGTTVTVSS** |
| **Etaracizumab_VK_Precursor** | **ELVMTQTPATLSVTPGDSVSLSCRASQSISNHLHWYQQKSHESPRLLIKYASQSISGIPSRFSGSGSGTDFTLSINSVETEDFGMYFCQQSNSWPHTFGGGTKLEIK** |
| **Etaracizumab_VK_Humanized** | **EIVLTQSPATLSLSPGERATLSCQASQSISNFLHWYQQRPGQAPRLLIRYRSQSISGIPARFSGSGSGTDFTLTISSLEPEDFAVYYCQQSGSWPLTFGGGTKVEIK** |
| **Talacoluzumab_VH_Precursor** | **EVQLQQSGPELVKPGASVKMSCKASGYTFTDYYMKWVKQSHGKSLEWIGDIIPSNGATFYNQKFKGKATLTVDRSSSTAYMHLNSLTSEDSAVYYCTRSHLLRASWFAYWGQGTLVTVSA** |
| **Talacoluzumab_VH_Humanized** | **EVQLVQSGAEVKKPGESLKISCKGSGYSFTDYYMKWARQMPGKGLEWMGDIIPSNGATFYNQKFKGQVTISADKSISTTYLQWSSLKASDTAMYYCARSHLLRASWFAYWGQGTMVTVSS** |
| **Talacoluzumab_VK_Precursor** | **DFVMTQSPSSLTVTAGEKVTMSCKSSQSLLNSGNQKNYLTWYLQKPGQPPKLLIYWASTRESGVPDRFTGSGSGTDFTLTISSVQAEDLAVYYCQNDYSYPYTFGGGTKLEIK** |
| **Talacoluzumab_VK_Humanized** | **DIVMTQSPDSLAVSLGERATINCESSQSLLNSGNQKNYLTWYQQKPGQPPKPLIYWASTRESGVPDRFSGSGSGTDFTLTISSLQAEDVAVYYCQNDYSYPYTFGQGTKLEIK** |
| **Rovalpituzumab_VH_Precursor** | **QIQLVQSGPELKKPGETVKISCKASGYTFTNYGMNWVKQAPGKGLKWMAWINTYTGEPTYADDFKGRFAFSLETSASTASLQIINLKNEDTATYFCARIGDSSPSDYWGQGTTLTVSS** |
| **Rovalpituzumab_VH_Humanized** | **QVQLVQSGAEVKKPGASVKVSCKASGYTFTNYGMNWVRQAPGQGLEWMGWINTYTGEPTYADDFKGRVTMTTDTSTSTAYMELRSLRSDDTAVYYCARIGDSSPSDYWGQGTLVTVSS** |
| **Rovalpituzumab_VK_Precursor** | **SIVMTQTPKFLLVSAGDRVTITCKASQSVSNDVVWYQQKPGQSPKLLIYYASNRYTGVPDRFAGSGYGTDFSFTISTVQAEDLAVYFCQQDYTSPWTFGGGTKLEIR** |
| **Rovalpituzumab_VK_Humanized** | **EIVMTQSPATLSVSPGERATLSCKASQSVSNDVVWYQQKPGQAPRLLIYYASNRYTGIPARFSGSGSGTEFTLTISSLQSEDFAVYYCQQDYTSPWTFGQGTKLEIK** |
| **Clazakizumab_VH_Precursor** | **QSLEESGGRLVTPGTPLTLTCTASGFSLSNYYVTWVRQAPGKGLEWIGIIYGSDETAYATWAIGRFTISKTSTTVDLKMTSLTAADTATYFCARDDSSDWDAKFNLWGQGTLVTVSS** |
| **Clazakizumab_VH_Humanized** | **EVQLVESGGGLVQPGGSLRLSCAASGFSLSNYYVTWVRQAPGKGLEWVGIIYGSDETAYATSAIGRFTISRDNSKNTLYLQMNSLRAEDTAVYYCARDDSSDWDAKFNLWGQGTLVTVSS** |
| **Clazakizumab_VK_Precursor** | **AYDMTQTPASVSAAVGGTVTIKCQASQSINNELSWYQQKPGQRPKLLIYRASTLASGVSSRFKGSGSGTEFTLTISDLECADAATYYCQQGYSLRNIDNAFGGGTEVVVK** |
| **Clazakizumab_VK_Humanized** | **AIQMTQSPSSLSASVGDRVTITCQASQSINNELSWYQQKPGKAPKLLIYRASTLASGVPSRFSGSGSGTDFTLTISSLQPDDFATYYCQQGYSLRNIDNAFGGGTKVEIK** |
| **Ligelizumab_VH_Precursor** | **QVQLQQSGAELMKPGASVKISCKTTGYTFSMYWLEWVKQRPGHGLEWVGEISPGTFTTNYNEKFKAKATFTADTSSNTAYLQLSGLTSEDSAVYFCARFSHFSGSNYDYFDYWGQGTSLTVSS** |
| **Ligelizumab_VH_Humanized** | **QVQLVQSGAEVMKPGSSVKVSCKASGYTFSWYWLEWVRQAPGHGLEWMGEIDPGTFTTNYNEKFKARVTFTADTSTSTAYMELSSLRSEDTAVYYCARFSHFSGSNYDYFDYWGQGTLVTVSS** |
| **Ligelizumab_VK_Precursor** | **DILLTQSPAILSVSPGERVSFSCRASQSIGTNIHWYQQRTDGSPRLLIKYASESISGIPSRFSGSGSGTEFTLNINSVESEDIADYYCQQSDSWPTTFGGGTKLEIK** |
| **Ligelizumab_VK_Humanized** | **EIVMTQSPATLSVSPGERATLSCRASQSIGTNIHWYQQKPGQAPRLLIYYASESISGIPARFSGSGSGTEFTLTISSLQSEDFAVYYCQQSWSWPTTFGGGTKVEIK** |
| **Crizanlizumab_VH_Precursor** | **QVQLQQSGPELVKPGALVKISCKASGYTFTSYDINWVKQRPGQGLEWIGWIYPGDGSIKYNEKFKGKATLTVDKSSSTAYMQVSSLTSENSAVYFCARRGEYGNYEGAMDYWGQGTTVTVSS** |
| **Crizanlizumab_VH_Humanized** | **QVQLVQSGAEVKKPGASVKVSCKVSGYTFTSYDINWVRQAPGKGLEWMGWIYPGDGSIKYNEKFKGRVTMTVDKSTDTAYMELSSLRSEDTAVYYCARRGEYGNYEGAMDYWGQGTLVTVSS** |
| **Crizanlizumab_VK_Precursor** | **DIVLTQSPASLAVSLGQRATISCKASQSVDYDGHSYMNWYQQKPGQPPKLLIYAASNLESGIPARFSGSGSGTDFTLNIHPVEEEDAATYYCQQSDENPLTFGTGTKLELK** |
| **Crizanlizumab_VK_Humanized** | **DIQMTQSPSSLSASVGDRVTITCKASQSVDYDGHSYMNWYQQKPGKAPKLLIYAASNLESGVPSRFSGSGSGTDFTLTISSLQPEDFATYYCQQSDENPLTFGGGTKVEIK** |
| **Mogamulizumab_VH_Precursor** | **EVQLVESGGDLMKPGGSLKISCAASGFIFSNYGMSWVRQTPDMRLEWVATISSASTYSYYPDSVKGRFTISRDNAENSLYLQMNSLRSEDTGIYYCGRHSDGNFAFGYWGRGTLVTVSA** |
| **Mogamulizumab_VH_Humanized** | **EVQLVESGGDLVQPGRSLRLSCAASGFIFSNYGMSWVRQAPGKGLEWVATISSASTYSYYPDSVKGRFTISRDNAKNSLYLQMNSLRVEDTALYYCGRHSDGNFAFGYWGQGTLVTVSS** |
| **Mogamulizumab_VK_Precursor** | **DVLMTQTPLSLPVSLGDQASISCRSSRNIVHINGDTYLEWYLQRPGQSPKLLIYKVSNRFSGVPDRFSGSGSGTDFTLKISRVEAEDLGVYYCFQGSLLPWTFGGGTRLEIR** |
| **Mogamulizumab_VK_Humanized** | **DVLMTQSPLSLPVTPGEPASISCRSSRNIVHINGDTYLEWYLQKPGQSPQLLIYKVSNRFSGVPDRFSGSGSGTDFTLKISRVEAEDVGVYYCFQGSLLPWTFGQGTKVEIK** |
| **Refanezumab_VH_Precursor** | **EIQLVQSGPELKKPGETNKISCKASGYTFTNYGMNWVKQAPGKGLKWMGWINTYTGEPTYADDFTGRFAFSLETSASTAYLQISNLKNEDTATYFCARNPINYYGINYEGYVMDYWGQGTLVTVSS** |
| **Refanezumab_VH_Humanized** | **QVQLVQSGSELKKPGASVKVSCKASGYTFTNYGMNWVRQAPGQGLEWMGWINTYTGEPTYADDFTGRFVFSLDTSVSTAYLQISSLKAEDTAVYYCARNPINYYGINYEGYVMDYWGQGTLVTVSS** |
| **Refanezumab_VK_Precursor** | **NIMMTQSPSSLAVSAGEKVTMSCKSSHSVLYSSNQKNYLAWYQQKPGQSPKLLIYWASTRESGVPDRFTGSGSGTDFTLTIINVHTEDLAVYYCHQYLSSLTFGTGTKLEIK** |
| **Refanezumab_VK_Humanized** | **DIVMTQSPDSLAVSLGERATINCKSSHSVLYSSNQKNYLAWYQQKPGQPPKLLIYWASTRESGVPDRFSGSGSGTDFTLTISSLQAEDVAVYYCHQYLSSLTFGQGTKLEIK** |

**
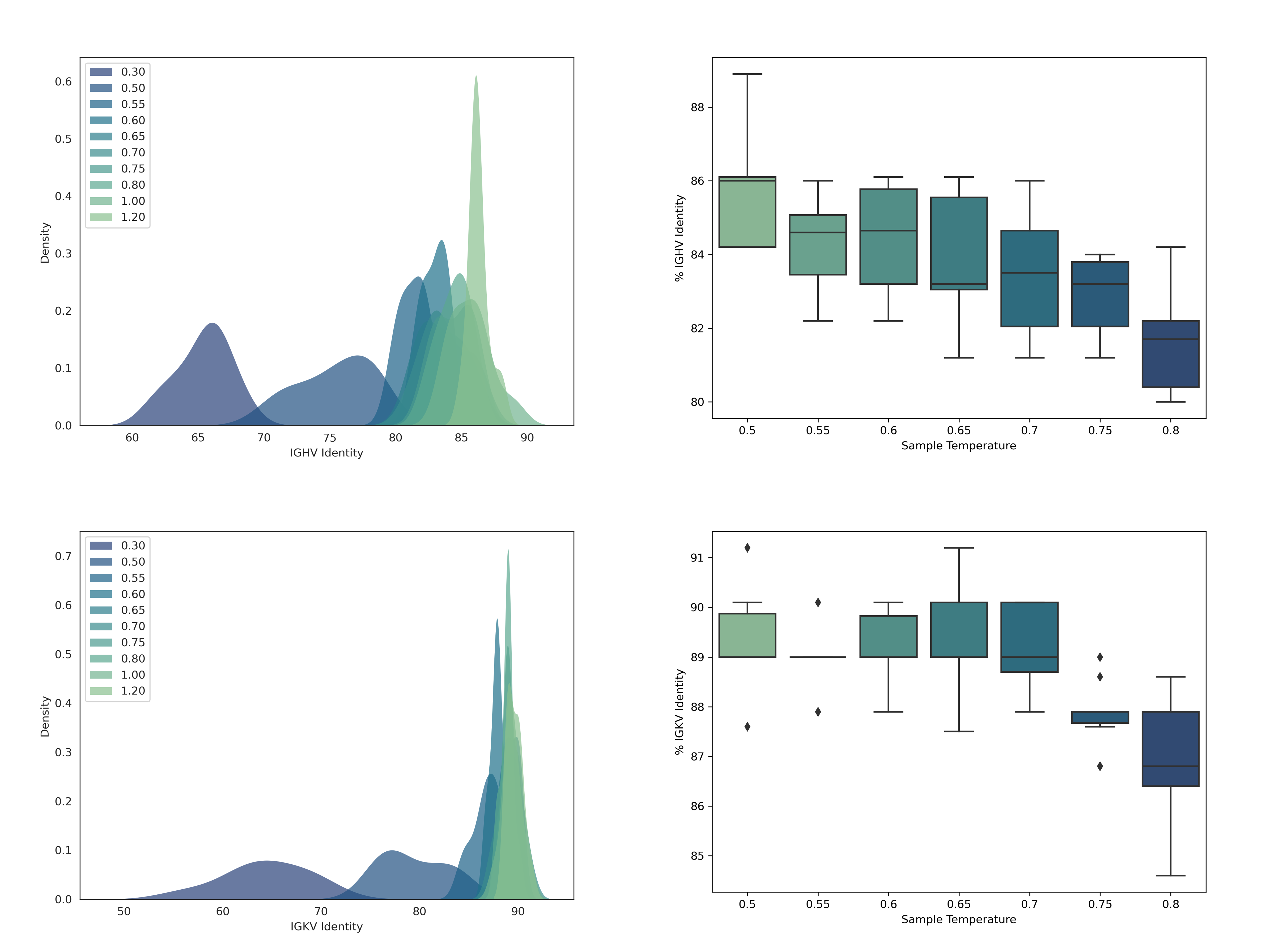
**

**Figure S1.** Temperature Sample in VH and VK. We used the IMGT DomainGapAlign (https://www.imgt.org/3Dstructure-DB/cgi/DomainGapAlign.cgi) tool to score the IGHV (IGKV) Identity between the sampled humanized sequences and human genes. Considering the impact of sampling temperature on the model, a low sampling temperature that could lead to excessive repetition of the sequence, while a high sampling temperature could result in unexpected outcomes. Therefore, we ultimately chosen a sampling temperature of 0.7 for heavy chains and 0.65 for light chains.

**
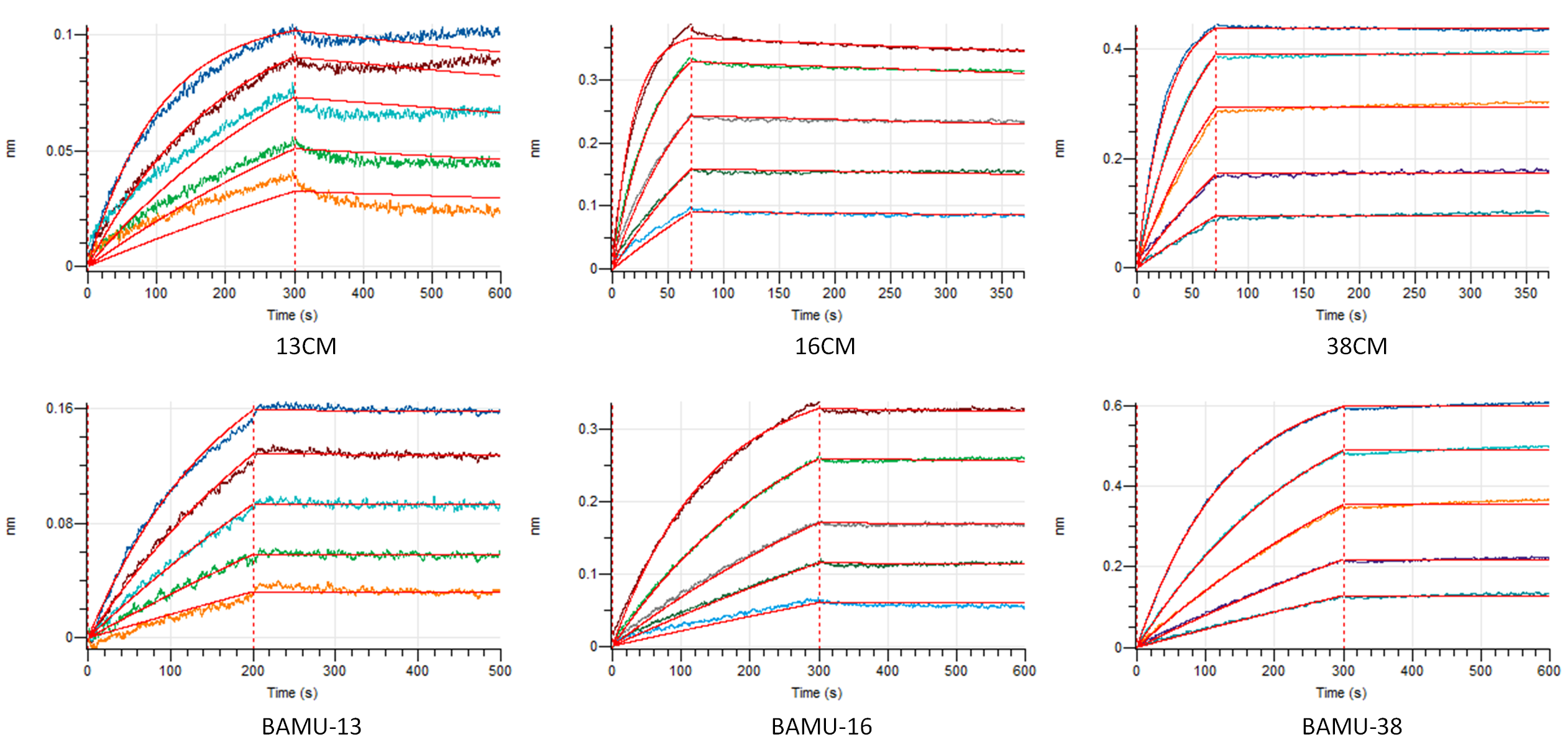
**

**Figure S2.** Variants 13,16 and 38 BLI results. This experiment was performed to measure the binding affinity of human IgG for protein (His tag) using Octet RED384. 0.03%Tween-20 in PBS, pH 7.2(GenScript, Lot. No. 20240718P0020) for baseline steps. The assay was performed at 30℃ and at 1000 rpm. Protein (His tag) was firstly immobilized onto NTA biosensor. Human IgG was applied as analyte for association and dissociation steps. All the data were processed using the Octet RED BLI Discovery version 12.2.2.26.
